# Phase 1/1b Studies of UCB0599, an Oral Inhibitor of α‐Synuclein Misfolding, Including a Randomized Study in Parkinson's Disease

**DOI:** 10.1002/mds.29170

**Published:** 2022-08-12

**Authors:** Johan Willem Smit, Peter Basile, Maria Key Prato, Laurent Detalle, François‐Xavier Mathy, Astrid Schmidt, Marianna Lalla, Massimiliano Germani, Coralie Domange, Anja‐Leona Biere, Massimo Bani, Stan Carson, Just Genius

**Affiliations:** ^1^ UCB Pharma Braine‐l'Alleud Belgium; ^2^ UCB Pharma Slough United Kingdom; ^3^ UCB Pharma Monheim am Rhein Germany; ^4^ UCB Pharma Brussels Belgium; ^5^ UCB Pharma Morrisville North Carolina USA; ^6^ Present address: Bergmapharm Consulting Verona Italy; ^7^ Present address: Genius Biotech Solutions, Ltd Victoria Malta

**Keywords:** Parkinson's disease, phase 1 clinical trial, α‐synuclein, misfolding inhibitor, disease modification

## Abstract

**Background:**

Parkinson's disease (PD) and its progression are thought to be caused and driven by misfolding of α‐synuclein (ASYN). UCB0599 is an oral, small‐molecule inhibitor of ASYN misfolding, aimed at slowing disease progression.

**Objective:**

The aim was to investigate safety/tolerability and pharmacokinetics (PK) of single and multiple doses of UCB0599.

**Methods:**

Safety/tolerability and PK of single and multiple doses of UCB0599 and its metabolites were investigated in two phase 1 studies in healthy participants (HPs), where food effect and possible interaction with itraconazole (ITZ) were assessed (UP0030 [randomized, placebo‐controlled, dose‐escalation, crossover study, N = 65] and UP0078 [open‐label study, N = 22]). Safety/tolerability and multi‐dose PK of UCB0599 were subsequently investigated in a phase 1b randomized, double‐blind, placebo‐controlled study of participants with PD (UP0077 [NCT04875962], N = 31).

**Results:**

Across all studies, UCB0599 displayed rapid absorption with linear, time‐independent PK properties; PK of multiple doses of UCB0599 were predictable from single‐dose exposures. No notable food‐effect was observed; co‐administration with ITZ affected UCB0599 disposition (maximum plasma concentration and area under the curve increased ~1.3‐ and ~2 to 3‐fold, respectively) however, this did not impact the safety profile. Hypersensitivity reactions were reported in UP0030 (n = 2) and UP0077 (n = 2). Treatment‐related adverse events occurred in 43% (UCB0599), and 30% (placebo) of participants with PD were predominantly mild‐to‐moderate in intensity and were not dose related.

**Conclusions:**

Seventy‐three HPs and 21 participants with PD received UCB0599 doses; an acceptable safety/tolerability profile and predictable PK support continued development of UCB0599 for the slowing of PD progression. A phase 2 study in early‐stage PD is underway (NCT04658186). © 2022 UCB Pharma. *Movement Disorders* published by Wiley Periodicals LLC on behalf of International Parkinson and Movement Disorder Society.

## Graphical abstract

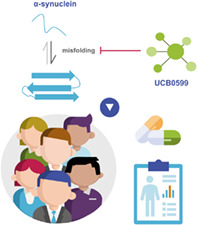

UCB0599, an oral small‐molecule inhibitor of alpha‐synuclein misfolding, aims to slow Parkinson's disease (PD) progression. During Phase 1/1b studies, UCB0599 demonstrated an acceptable safety/tolerability profile and predictable pharmacokinetics in healthy participants, including older individuals, and participants with PD. These data have informed the further clinical development of UCB0599.

Parkinson's disease (PD) is a progressive neurodegenerative disorder that presents with a spectrum of nonmotor and motor symptoms.^1^, ^2^ Genetic studies point to a causal role of α‐synuclein (ASYN) in PD, as mutations and duplications of the gene coding for ASYN (*SNCA)* have been linked to rare, inherited forms of PD.^3^ Misfolding of ASYN is considered a key process in the pathogenesis of PD.^4^, ^5^ Monomeric ASYN self‐assembles into oligomeric units, which can grow into insoluble, macroscopic, β‐sheet‐rich intracellular structures termed Lewy bodies and Lewy neurites.^1^, ^5^ Identification of Lewy bodies and degeneration of the substantia nigra at autopsy are the gold standard for diagnosis of PD,^1^ and the process of pathological misfolding (leading to the formation of Lewy bodies) is associated with further deleterious changes, including, but not limited to: neuroinflammation, lysosomal dysfunction and oxidative stress, and ultimately neuronal death that underlies disease progression,^6^, ^7^, ^8^ suggesting ASYN is a promising therapeutic target in PD.^9^, ^10^, ^11^

Current treatments for PD provide only partial symptomatic relief but do not delay PD progression.^1^, ^12^, ^13^ Treatment of late‐stage PD can be complex as increasing doses of dopaminergic therapies (which commonly cause side effects, including “wearing‐off” effects and nonmotor/motor fluctuations) are required to treat progressing symptoms.^1^, ^2^, ^14^ This highlights the immense unmet need for disease‐modifying treatments that target the underlying pathology of PD and offer the potential to reduce or stop neurodegeneration and disease progression.^13^ UCB0599 (the R‐enantiomer of NPT200‐11)^15^ is a brain‐penetrant, orally available small molecule that disrupts the first step in the ASYN aggregation cascade by preventing misfolding and the formation of ASYN aggregates on lipid membranes.^11^, ^16^ In a preclinical study, chronic in‐vivo dosing with UCB0599 in ASYN transgenic mice decreased aggregated ASYN levels and improved functional motor endpoints associated with PD.^16^

Here, we report the results of two phase 1 studies (UP0030 and UP0078) in healthy participants (HPs), assessing the effect of food and itraconazole (ITZ) on UCB0599 disposition; we subsequently report the findings of a phase 1b study assessing safety/tolerability and pharmacokinetics (PK) of UCB0599 in participants with PD (UP0077, NCT04875962). UP0030 and UP0077 are single ascending dose (SAD) and/or multiple ascending dose (MAD) studies completed to ensure the safety of study participants; once an acceptable safety profile at lower doses is established, then higher doses are administered.^17^

## Patients and Methods

### Ethical Conduct

Study protocols, amendments, and participant‐informed consent were reviewed by relevant ethics committees; ethical conduct details, sample size, randomization, and adverse events of special interest (AESI) for each study are included in the Supporting Information.

### UP0030

#### Study Design and Eligibility

UP0030 was a two‐part, phase 1, SAD and MAD study to evaluate the safety/tolerability, PK, potential effect of food, and drug–drug interactions following ITZ, a strong cytochrome P450 3A4 (CYP3A4) inhibitor, on orally administered UCB0599 in HPs. Part 1 (Fig. S1A) was a randomized, subject‐ and investigator‐blind, placebo‐controlled study in healthy older participants (HOP). Part 2 (Fig. S1B) was an open‐label, two‐way crossover study in HPs.

Part 1 included five cohorts (1, 2, 1b, 2b, and 3) and evaluated SADs (SAD: 90, 180, 360, and 450 mg) and MADs of UCB0599 (MAD: 180 and 360 mg/day). In Part 2, participants received 360 mg of UCB0599 on three occasions, with a washout period of 7 to 14 days between doses. The first two doses were administered as a two‐way crossover (under fasting/fed or fed/fasting conditions), and the third dose was administered to both arms under fasted conditions with 200 mg of ITZ (once daily, days −3 to 2) to assess potential drug–drug interactions.

Eligible participants were aged ≥18 years (part 1, 55–75 years for HOP; part 2, <55 years) with a body mass index (BMI) of 18–32 kg/m^2^ and a body weight of ≥50 kg. The study was conducted at a phase 1 unit in Germany.

#### Objectives and Variables

The primary objective was to evaluate the safety and tolerability of UCB0599 after the administration of single and multiple doses in HOP. The secondary objectives are to evaluate the PK of UCB0599 and its desmethyl and N‐oxide metabolites after the administration of single and multiple doses in HOP and to evaluate the PK, safety, and tolerability of UCB0599 in HPs when given a single dose alone, with food or with ITZ. In part 1, the collection of cerebrospinal fluid (CSF) to assess UCB0599 concentration was completed ~2 hours after the administration at the following time points: day 1: all SAD, day 14: 180 mg/day (cohort 1), and day 21: 180 mg/day (cohort 1b).

Safety variables included the incidence of any treatment‐emergent adverse event (TEAE), changes in vital signs, laboratory assessments, 12‐lead electrocardiogram (ECG) and Holter ECG assessment, and the Columbia Suicide Severity Rating Scale (C‐SSRS). PK variables included maximum plasma concentration (*C*_max_), time of maximum concentration (*t*_max_), AUC (area under the plasma concentration–time curve) from time 0 to last quantifiable concentration (AUC_0–*t*_), AUC from time 0 to infinity (AUC_0–inf_), apparent total body clearance, apparent volume of distribution, and apparent elimination half‐life (*t*_1/2_).

### UP0078

#### Study Design and Eligibility

UP0078 was a phase 1, open‐label study to evaluate the potential effect of food on the bioavailability of UCB0599 and to test the potential effect of ITZ on the disposition of UCB0599 in HPs. A randomized, two‐way crossover design with a third fixed‐treatment period was used (Fig. S2).

Participants received a single 180 mg dose of UCB0599 on three separate occasions, with a washout period of ≥7 days between doses. The first dose was administered under fasted conditions, the second under fed conditions, and the third under fasted conditions co‐administered with ITZ (200 mg once daily, days 15–22).

Eligible participants were aged 18 to 55 years and had a BMI of 18 to 30 kg/m^2^ and a body weight of ≥50 kg. The study was conducted at a phase 1 unit in the United Kingdom.

#### Objectives and Variables

The primary objectives were to assess the potential effect of food intake (standard high‐fat, high‐calorie meal) on single‐dose PK of UCB0599 and to evaluate and compare the single‐dose PK of UCB0599 in the presence or absence of ITZ. The secondary and exploratory objectives were to evaluate the safety and tolerability of UCB0599 under fasted and fed conditions, and with/without concomitant administration of ITZ; to evaluate UCB0599 metabolite plasma PK in the presence or absence of ITZ; and to assess the potential effect of food intake on the single‐dose PK of UCB0599 metabolites.

Primary PK variables were AUC_0–*t*_, AUC_0–inf_, *C*_max_, and *t*_max_. Primary safety variables were the incidence of TEAEs and serious TEAEs. Other safety variables included changes in vital signs, changes in laboratory data, changes in 12‐lead ECG assessment, and physical examination findings.

### UP0077

#### Study Design and Eligibility

UP0077 (NCT04875962) was a randomized, double‐blind, placebo‐controlled phase 1b study evaluating safety, tolerability, and PK of multiple doses of UCB0599 in HPs and participants with PD. Clinical research units were encouraged to use their local network to recruit. Due to prioritization of participants with PD, and good recruitment in this group, no HPs were recruited to UP0077. Participants were evaluated in sequential cohorts (Fig. S3). Cohort 1 received UCB0599 180 mg/day (90 mg twice daily) or placebo for 28 days, and cohort 2 received UCB0599 360 mg/day (180 mg twice daily) or placebo for 28 days (randomized 2:1, UCB0599:placebo, for both cohorts). Participants stayed at phase 1 clinical research units with inpatient capabilities (a regulatory authority request), for the entire treatment period and 48 hours after the last dose, to maximize the likelihood of detecting early safety signals. A final safety visit was conducted 14 days after the last dose and discharged from the research units.

Eligible participants were aged 40 to 80 years, with a clinical diagnosis of PD (based on the UK Parkinson's Disease Society Brain Bank clinical diagnostic criteria),^18^ with bradykinesia and muscular rigidity and/or resting tremor, and with a Hoehn and Yahr stage 1 to 3. If participants were receiving treatment for PD, the regimen had to be stable for ≥4 weeks prior to study entry, and it was expected that it would remain stable for the duration of this study. Participants with PD were required to have no evidence of brain abnormalities on magnetic resonance imaging or computed tomography that could cause symptomatic Parkinsonism or other pathologies that could interfere with their ability to adhere to the study protocol. BMI was 18 to 32 kg/m^2^, and body weight was >50 kg. The study was conducted at six sites in the USA.

#### Objectives and Variables

The primary objective was to evaluate safety and tolerability of UCB0599 in participants after multiple‐dose administrations (180 and 360 mg/day). The secondary objective was to evaluate the PK of UCB0599 after single‐ and multiple‐dose administration. Exploratory objectives included PK evaluation of UCB0599, and its desmethyl and N‐oxide metabolites, after single and multiple dosing, and evaluation of potential interconversion of UCB0599 into its S‐enantiomeric form following multiple‐dose administration.

The primary safety variable was incidence of any TEAE from baseline to week 7. Other safety variables included changes in vital signs (heart rate, blood pressure, respiratory rate, and body temperature), changes in laboratory data (hematology, clinical chemistry, functional immunology parameters, and urinalysis), changes in 12‐lead ECG assessment, physical examination findings including detailed neurological examinations, and the C‐SSRS. The PK variables were *C*_max_, *t*_max_, area under the plasma concentration–time curve 0 to 12 hours (AUC_0–12h_), and AUC for the dosing interval at steady state (AUC_Ƭ_), measured on day 1 (pre‐dose, <12 hours post‐dose) and/or day 28 (pre‐dose, <48 hours post‐dose).

#### Statistical Analyses

Statistical evaluations for all three studies were performed using SAS version 9.4 (SAS, Cary, NC, USA), and PK data were analyzed using Phoenix WinNonlin software (Certara, Princeton, NJ, USA). Continuous variables were summarized with number of participants, mean, standard deviation, median, minimum and maximum, and confidence intervals (CI) for the mean. Categorical variables were summarized with frequency counts and percentages. Coefficient of variation, geometric mean (GeoMean), and 95% CI were also presented in the descriptive statistics for PK data.

## Results

### Participant Disposition

#### UP0030: Two‐Part Phase 1 Study Evaluating Safety/Tolerability, PK, and the Effect of Food and ITZ on UCB0599

Sixty‐five HPs were randomized to receive UCB0599 or placebo, between March 8, 2017, and May 18, 2018; 22 participants completed, and 43 discontinued (most discontinuations [n = 37 (56.9%)] were due to study termination; Fig. S4). Baseline demographics are presented in Table 1.

**TABLE 1:** Participant demographics and baseline disease characteristics for participants with PD

	UP0030 (phase 1 study—HPs)				UP0078 (phase 1 study—HPs)	UP0077 (phase 1b study—participants with PD)	
	Part 1		Part 2				
	All UCB0599 N = 45	All Placebo N = 14	Fasted UCB0599 n = 3	Fed UCB0599 n = 3	All UCB0599 N = 22	All UCB0599 N = 21	Placebo N = 10
Median (range) age (y)	65.0 (55–74)	64.0 (55–73)	25.0 (18–53)	41.0 (35–47)	38.0 (21–52)	66.0 (47–80)	68.5 (46–76)
Female, n (%)	14 (31.1)	5 (35.7)	0	0	2 (9.1)	8 (38.1)	2 (20.0)
Median (range) BMI (kg/m^2^)	26.40 (20.6–31.4)	25.50 (20.8–30.8)	22.50 (22.0–23.6)	25.70 (23.2–30.1)	25.15 (20.0–28.7)	27.60 (20.3–32.0)	23.10 (20.1–30.5)
Race, n (%)							
Asian	0	0	0	0	2 (9.1)	0	0
Black or African American	0	0	0	0	0	3 (14.3)	1 (10.0)
White	45 (100)	14 (100)	3 (100)	3 (100)	20 (90.9)	18 (85.7)	9 (90.0)
Median (range) MDS‐UPDRS score	NA	NA	NA	NA	NA	64.0 (17–111)	63.0 (41–99)
Median (range), Hoehn and Yahr stage at screening	NA	NA	NA	NA	NA	2.0 (2–3)	2.0 (2–3)
Median (range) duration of PD (mo)	NA	NA	NA	NA	NA	66.0 (13–179)	101.0 (35–227)
Currently on PD medication, n (%)	NA	NA	NA	NA	NA	19 (90.5)	10 (100.0)

Baseline characteristics were generally well balanced across the groups within each study.

Abbreviations: HPs, healthy participants; PD, Parkinson's disease; BMI, body mass index; MDS‐UPDRS, Movement Disorders Society‐Unified Parkinson's Disease Rating Scale; n/N, number of participants; NA, not applicable.

#### UP0078: Phase 1 Study Evaluating the Effect of Food and ITZ on UCB0599

Between October 29, 2019, and January 9, 2020, 22 HPs were randomized to receive UCB0599, and all completed the study (Fig. S5). Baseline demographics are presented in Table 1.

#### UP0077: Phase 1b Study Evaluating Safety/Tolerability and Multi‐dose PK of UCB0599

Between May 6, 2019, and February 19, 2020, 31 participants with PD were randomized to receive UCB0599 or placebo (Fig. S6). A total of 29 participants (93.5%) completed the study, median age: 66 years (range: 46–80); most participants were male (67.7%). Demographics and baseline PD characteristics were generally consistent across groups (Table 1), although disease duration and severity (based on mean Movement Disorder Society‐Unified Parkinson's Disease Rating Scale) were lower in the UCB0599 180 mg/day group compared with the placebo and UCB0599 360 mg/day groups. All participants with PD used concomitant medication at baseline: medications in the anatomical main group of the nervous system were reported by most participants (96.8%); common concomitant medications and medical history are presented in Table S1.

### Safety

#### UP0030: Two‐Part Phase 1 Study Evaluating Safety/Tolerability, PK, and the Effect of Food and ITZ on UCB0599

The primary objective for UP0030 was to evaluate the safety and tolerability of UCB0599 after single and multiple doses in HOP. In Part 1, a similar percentage of TEAEs was reported in the total UCB0599 (30 participants [66.7%] reported 89 TEAEs) and placebo groups (9 participants [64.3%] reported 27 TEAEs) (Table 2). The most common TEAEs are presented in Table S2. Most TEAEs were mild‐to‐moderate in intensity; severe TEAEs were reported by 3 participants (6.7%) in the total UCB0599 group (blood creatine phosphokinase increased, presyncope, hypertension [1 event each]) and 2 (14.3%) in the placebo group (presyncope [2 events]). Two participants (4.4%) reported serious TEAEs, and 2 additional TEAEs were upgraded to serious by UCB (4 participants in total). There were 6 discontinuations due to TEAEs: 5 participants (11.1%) discontinued the study in the total UCB0599 group (drug hypersensitivity [2 participants], intervertebral disc disorder, hypertension, and atrial fibrillation [1 event per participant]), and 1 participant (7.1%) discontinued in the placebo group (ventricular tachycardia [resulting in a study hold]).

**TABLE 2:** Safety overview

n (%) [#]	UP0030 (phase 1 study—HPs)						
	Part 1		Part 2		UP0078 (phase 1 study—HPs)	UP0077 (phase 1b study—participants with PD)	
	All UCB0599 N = 45	All Placebo N = 14	Fasted UCB0599 n = 3*	Fed UCB0599 n = 3*	UCB0599 N = 22	All UCB0599 N = 21	Placebo N = 10
Any TEAEs	30 (66.7) [89]	9 (64.3) [27]	0	1 (33.3) [1]	6 (27.3) [8]	17 (81.0) [55]	7 (70.0) [19]
Serious TEAEs^a^	2 (4.4) [2]	0	0	0	0	1 (4.8) [1]	1 (10.0) [1]
Discontinuations due to TEAEs	5 (11.1) [5]	1 (7.1) [1]	0	0	0	1 (4.8) [2]	1 (10.0) [2]
Drug‐related TEAEs	14 (31.1) [28]	3 (21.4) [7]	0	0	1 (4.5) [1]	9 (42.9) [31]	3 (30.0) [8]
Severe TEAEs	3 (6.7) [4]	2 (14.3) [2]	0	0	0	4 (19.0) [4]	1 (10.0) [1]
TEAEs of special interest^b^	NA	NA	NA	NA	0	2 (9.5) [2]	0
Deaths	0	0	0	0	0	0	0

Of the doses assessed, UCB0599 demonstrated an acceptable safety/tolerability profile in HPs and participants with PD with age‐related comorbidities; the majority of AEs were mild‐to‐moderate in intensity, and no increase in the frequency/severity of TEAEs was observed with increasing doses of UCB0599.

Abbreviations: HPs, healthy participants; PD, Parkinson's disease; TEAE, treatment‐emergent adverse event; HSR, hypersensitivity reaction; n, number of participants reporting a TEAE in that category.

[#] Number of TEAEs.

* Due to early termination of UP0030, n = 3 participants were recruited to each group in part 2 of the study.

^a^ UP0077–UCB0599 360 mg/day: listed as kidney injury and updated to chronic renal failure after drug washout period, which was severe in intensity and considered related to UCB0599. On review, this participant's renal laboratory abnormalities were noted not to have reached standard Kidney Disease: Improving Global Outcomes criteria for either acute or chronic renal failure. Placebo: syncope and a concurrent TEAE of ECG QT prolongation resulting in discontinuation from the study. UP0030–UCB0599 180 mg: HSR, considered of moderate intensity, related to UCB0599, and resulted in discontinuation from the study; UCB0599 360 mg: diverticulitis, of moderate intensity, 39 days after the last dose and was not related to UCB0599.

^b^ TEAEs of special interest were as follows: UCB0599 360 mg: type IV hypersensitivity reaction; UCB0599 180 mg/day: hypersensitivity (later clarified as “nausea, lightheaded, and dizziness”). One additional TEAE of rash (urticaria preceding “moderate lip swelling”) was reported in the 360 mg/day group and led to discontinuation from the study.

In part 2, 1 TEAE was reported by 1 participant (33.3%) in the fed UCB0599 360 mg group. This TEAE was non‐serious, did not lead to discontinuation from the study, and was not considered related to UCB0599.

There were no deaths in the UP0030 study. Overall, no dose effect on the number or severity of TEAEs was observed, and there were no discernible UCB0599 treatment‐related patterns for any vital signs, ECG tracings, or laboratory findings. There were 2 cases of hypersensitivity reaction (HSR): both in the 180 mg dose group, both moderate in intensity and considered related to UCB0599. Each participant received oral antihistamines, and the HSRs resolved within 3 days. UCB decided to terminate UP0030 prior to completion of assessments of the UCB0599 360 mg/day cohort in parts 1 and 2 as a precautionary measure and to develop an additional protocol to closely monitor safety events, particularly potential HSRs.

#### *UP0078* : Phase 1 Study Evaluating the Effect of Food and ITZ on UCB0599

Six participants (27.3%) reported 8 TEAEs, no TEAEs resulted in a discontinuation from the study, and there were no serious or severe TEAEs (Table 2). No TEAEs by preferred term were reported by more than 1 participant. One TEAE (4.5%) was considered related to UCB0599 (oropharyngeal pain), and 2 (9.1%) were considered related to ITZ (pain in extremity and oropharyngeal pain). No clinically significant abnormalities were observed in vital signs, laboratory evaluations, physical examinations, or ECG findings. Safety and tolerability of UCB0599 were similar under fasted/fed conditions, and the safety profile of UCB0599 with concomitant ITZ was similar to UCB0599 alone. There were no AESI; no TEAEs led to discontinuation from the study, and there were no deaths.

#### UP0077: Phase 1b Study Evaluating Safety/Tolerability and Multi‐dose PKs of UCB0599

The primary objective of UP0077 was to evaluate safety and tolerability of UCB0599 in participants after multiple‐dose administration (180 and 360 mg/day). In a population of older participants with PD, 17 (81%) in the total UCB0599 group reported 55 TEAEs, and 7 (70%) in the placebo group reported 19 TEAEs (Table 2); the most common TEAEs are listed in Table S3. There were 2 discontinuations due to TEAEs, 1 in the placebo group and 1 in the UCB0599 360 mg/day group. In sum, 1 serious TEAEs were reported, 1 from a participant in the placebo group (syncope and concurrent ECG QT prolongation, resulting in discontinuation from the study) and 1 from a participant in the UCB0599 360 mg/day group (kidney injury, updated to chronic renal failure after drug‐washout period). Initially the investigator reported this serious TEAE as acute renal failure, considered to be related to UCB0599; however, 112 days after the last dose of UCB0599, the investigator updated the report to chronic renal failure because abnormalities were still present. A review by the sponsor showed that this participant's renal laboratory abnormalities were found not to have reached standard criteria for either acute or chronic renal failure.^19^ One participant from the UCB0599 360 mg/day group also reported a serious adverse event, which was not treatment emergent, of non‐cardiac chest pain (diagnosed as gastroesophageal reflux disorder) 16 days after the last dose of UCB0599; this was not considered related to UCB0599 by the investigator.

TEAEs considered by the investigator to be related to the study medication were reported by 9 participants (43%, reporting 31 TEAEs) in the total UCB0599 group and 3 (30%, reporting 8 TEAEs) in the placebo group (Table S4). The majority of TEAEs were mild‐to‐moderate in intensity; severe TEAEs were reported by 4 participants in the UCB0599 total group (headache [2 events], acute kidney injury, and nasal congestion [1 event each]) and 1 participant in the placebo group (syncope). No increase in severity of TEAEs was observed with increasing doses of UCB0599.

Two AESI were reported, a type IV HSR reported in 1 participant (27 days after receiving the first dose of 360 mg/day, classified as mild and considered not related to UCB0599 by the investigator) and 1 case of hypersensitivity (3 days after receiving the first dose of 180 mg/day; later clarified by the investigator as “nausea, lightheaded, and dizziness”). One additional TEAE, rash, was reported in the 360 mg/day group and led to discontinuation from the study. This event consisted of urticaria (occurring after 12 days of UCB0599 dosing) preceding “moderate lip swelling”; a biopsy did not confirm whether the rash was a type I or IV HSR (Stevens–Johnson‐type reaction was excluded as no perivascular polymorphonuclear leucocyte infiltrates were present).

Overall, no consistent or clinically relevant treatment‐related patterns were observed for any laboratory findings, vital signs, or ECG findings during this study (including QT interval corrected using Fridericia's correction QTcF intervals [Fig. S7] and plasma creatinine trajectories for cohort 1 [Fig. S8a] and cohort 2 [Fig. S8b] participants), and no deaths were reported. Safety results are consistent with earlier phase 1 studies (UP0078 and UP0030) in HPs.

### Pharmacokinetics

#### UP0030: Two‐Part Phase 1 Study Evaluating Safety/Tolerability, PK, and the Effect of Food and ITZ on UCB0599

Four SADs of UCB0599 (range: 90–450 mg) were explored. UCB0599 PK results showed a dose‐proportional increase in AUC (GeoMean 90 mg: 3225 h*ng/mL; 450 mg: 17,040 h*ng/mL) and *C*_max_ (GeoMean 90 mg: 374 ng/mL; 450 mg: 1806 ng/mL), with rapid absorption that did not alter with increasing doses (*t*_max_ range: 1.5–2.0 hours). The apparent elimination half‐life was similar across dose groups (GeoMean range: 10.6–13.0 hours). Plasma exposures of the desmethyl metabolite over time were lower, and the apparent elimination half‐life was longer, compared with UCB0599 (GeoMean *t*_1/2_ ranges: 16.8–17.5 and 10.6–13.0 hours, respectively). PK parameters for the N‐oxide metabolite were similar to those for UCB0599.

Following 180 mg/day of UCB0599 on days 7, 14, and 21, concentration–time profiles were comparable, indicating plasma steady‐state concentration was achieved, on average, by day 7. Median *t*_max_ values after multiple doses (UCB0599 180 mg/day) were 1.5–3.0 hours, similar to those obtained after single‐dose administration. PK properties of UCB0599 were linear across the tested dose range, meaning multiple‐dose exposures were predictable from single‐dose exposures. The PK properties of the desmethyl and N‐oxide metabolites were similar after multiple doses of UCB0599 from days 7 to 21.

**FIG 1:**
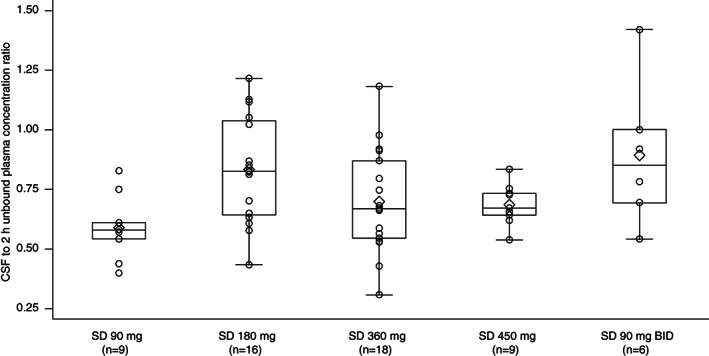
Ratio of cerebrospinal fluid (CSF) to estimated unbound UCB0599 plasma concentration 2 hours after drug intake, by treatment group in UP0030 (phase 1 study in healthy participants [HPs]). Unbound plasma concentration is estimated by multiplying concentration by 0.011 (fraction unbound). Per protocol, plasma concentrations were measured at 2 hours post‐dose, shortly before CSF sampling. The symbol ◊ indicates the mean values of the calculated ratios in the boxplots. The concentrations of UCB0599 in the CSF increased linearly with increasing doses. BID, twice daily; CSF, cerebrospinal fluid; HPs, healthy participants; SD, single dose.

CSF sampling was closely timed to the 2‐hour post‐dose plasma collection. UCB0599 concentrations in the CSF showed a linear increase with increasing dose (GeoMean SAD: 90 mg: 2040 pg/mL; 450 mg: 11,800 pg/mL; MAD: 180 mg/day, day 14: 1260 pg/mL; 180 mg/day, day 21: 4880 pg/mL). Calculated UCB0599 unbound‐plasma concentrations were compared with CSF UCB0599 concentrations for the single‐ and multiple‐dose groups. The resulting CSF to unbound UCB0599 ratios were calculated; median ratios were 0.6 to 0.9 and indicated no dose‐dependent trend (Fig. 1). Concentrations of the desmethyl metabolites in the CSF were low/not measurable (~20 times lower than UCB0599 concentrations 2 hours post‐dose), suggesting low penetration into the brain, which is in agreement with the high apical efflux ratio (~9) observed in a Caco‐2 cell assay (data not shown). N‐oxide metabolites were below the limit of quantification in the CSF.

#### *UP0078* : Phase 1 Study Evaluating the Effect of Food and ITZ on UCB0599

The primary objectives of UP0078 were to assess the potential effect of food intake on single‐dose PK of UCB0599 and to evaluate and compare the effect of ITZ on single‐dose PK of UCB0599. The *C*_max_ and AUC GeoMean for UCB0599 were comparable under fed and fasted conditions (Table S5). The geometric least squares mean (GeoLSM [90% CI]) fed/fasted ratios for *C*_max_ and AUC_(0–*t*)_ were 104.5% (90.79–120.2) and 108.9% (104.4–113.7), respectively, indicating no meaningful food effect on UCB0599 absorption. For desmethyl and N‐oxide metabolites, the 90% CIs for *C*_max_ were slightly outside the 80% to 125% range; however, findings were not clinically significant, indicating no notable effect of food on *C*_max_ and AUC_(0–*t*)_ (Fig. S9).

Food consumption caused a delay in UCB0599 *t*_max_, with a median difference of 1.5 hours (90% CI: 1.0–2.0, Hodges–Lehmann estimate) between fed and fasted conditions; this delay had minimal impact on the absorption phase, exposure, peak plasma concentrations, and elimination phases for the UCB0599 parent molecule. Similar delays in the *t*_max_ were also observed for the metabolites.

Co‐administration of UCB0599 and ITZ resulted in increased (peak) plasma concentrations for UCB0599 (Fig. S9); the effects observed on metabolite concentration–time profiles were consistent with observations for the UCB0599 parent molecule. The point estimates for the GeoLSM (90% CI) ratios of UCB0599 + ITZ versus UCB0599 fasted were 130.4 (113.9–149.4) for *C*_max_ and 278.7% (262.1–296.3) for AUC_(0–*t*)_, demonstrating an increase in these parameters for the UCB0599 + ITZ group. The median difference in *t*_max_ between UCB0599 + ITZ and UCB0599 fasted was 0.5 hour (90% CI: 0–1.0), based on Hodges–Lehmann estimate (Fig. S9). These findings were in line with predicted patterns, as ITZ concentrations reached levels consistent with CYP3A inhibition.^20^

#### UP0077: Phase 1b Study Evaluating Safety/Tolerability and Multi‐dose PK of UCB0599

UCB0599 plasma concentration profiles after oral administration of 180 and 360 mg/day UCB0599 were analyzed; the compound demonstrated dose‐proportional increases in AUC and *C*_max_ (Table 3). Rapid absorption was observed, and *t*_max_ was similar with increasing doses (Table 3).

**TABLE 3:** UP0077 (phase 1b study in participants with PD) pharmacokinetic parameters of UCB0599 and its metabolites

			180 mg/day dose, n = 7			360 mg/day dose, n = 14		
	Statistics	Day	UCB0599	Desmethyl metabolite	N‐oxide metabolite	UCB0599	Desmethyl metabolite	N‐oxide metabolite
AUC (h*ng/mL)	Geometric mean (CV%)	1	2233 (27.8)	77.01 (55.6)	2170 (42.1)	3387 (48.7)	78.49 (50.7)	2359 (43.9)
		28	3736 (42.3)	184.6 (67.1)	3914 (48.4)	7652 (28.1)^a^	315.5 (55.2)^a^	5777 (38.8)^a^
*C*_max_ (ng/mL)	Geometric mean (CV%)	1	417.3 (36.6)	10.22 (48.2)	287.9 (40.3)	662.1 (53.0)	11.56 (51.2)	320.8 (36.1)
		28	670.8 (43.1)	22.15 (65.2)	501.9 (45.2)	1273 (33.4)^a^	34.34 (58.1)^a^	652.2 (41.7)^a^
*t*_max_ (h)	Median (range)	1	2.0 (1.0–2.85)	1.980 (1.0–8.0)	3.00 (1.8–4.0)	2.0 (1.0–6.13)	2.0 (0.5–7.9)	3.0 (1.0–7.9)
		28	2.0 (1.5–4.0)	1.550 (1.0–4.0)	2.750 (1.5–4.0)	2.0 (1.0–6.12)^a^	1.6 (1.0–8.0)^a^	3.1 (1.5–8.0)^a^
Half‐life (h)	Geometric mean (CV%)	28	10.73 (32.8)	15.11 (26.4)	13.84 (43.5)	13.08 (23.9)^a^	18.70 (22.7)^a^	13.84 (27.7)^a^
Fraction unbound (%)	Geometric mean (CV%)	1	1.1 (31.0)	–	–	1.0 (32.2)^a^	–	–
		28	1.2 (37.1)			1.0 (25.4)^b^		

The PK of multiple doses of UCB0599 were predictable from single‐dose exposures. *C*_max_ and AUC demonstrated dose‐proportional increases, and UCB0599 fraction unbound was constant between dose groups.

Abbreviations: PD, Parkinson's disease; AUC, area under the plasma concentration–time curve from 0 to 12 hours (day 1) and for the dosing interval at steady state (day 28); *C*_max_, maximum plasma concentration; CV, coefficient of variation; *t*_max_, time of maximum concentration; PK, pharmacokinetic.

^a^ n = 13 for these PK parameters.

^**b**^ n = 10 for this PK parameter. PK‐per protocol set.

**FIG 2:**
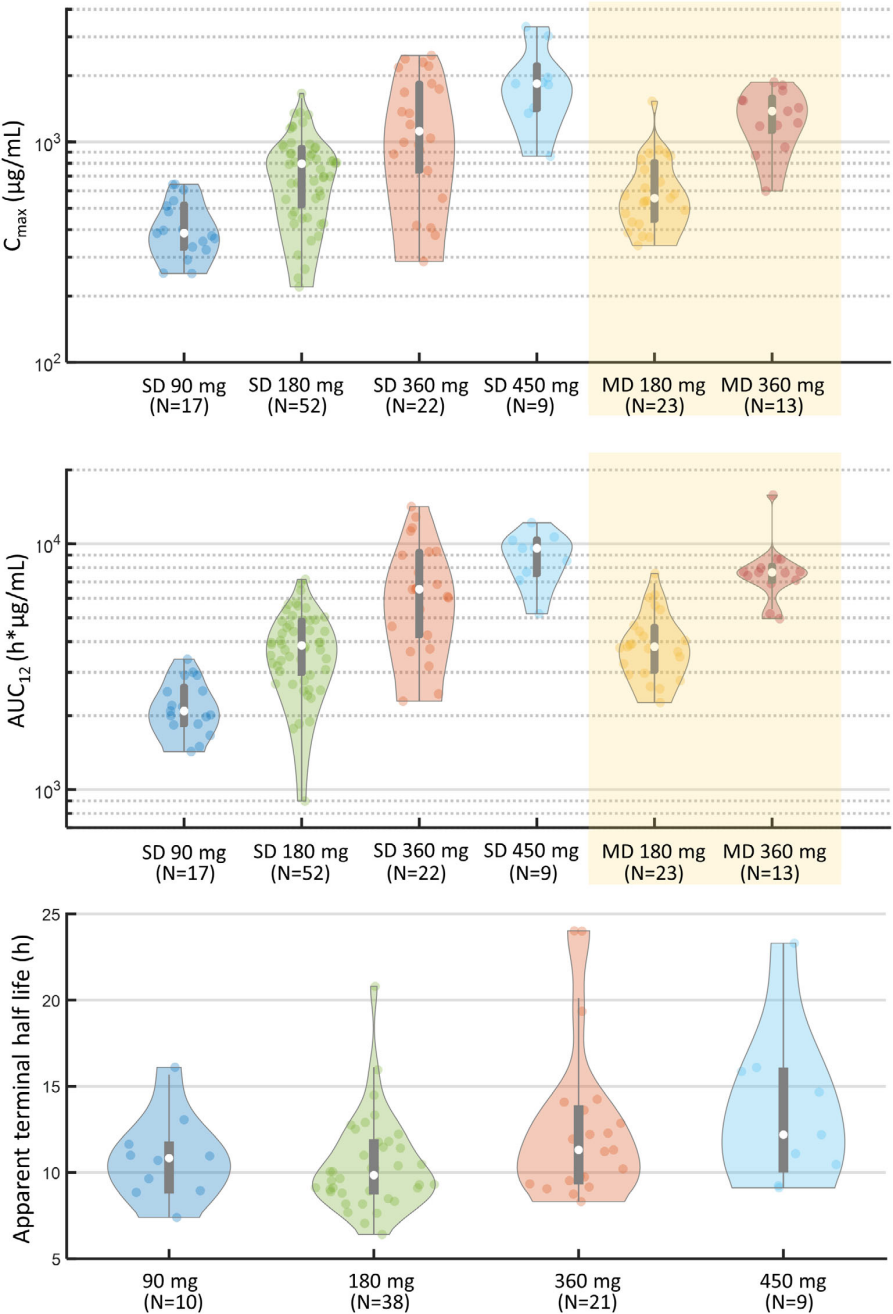
Overview of PK (pharmacokinetic) data from the UCB0599 phase 1/1b studies. Violin plots show the distribution of data in each group. Medians (white spots), interquartile range delimited by 25th and 75th percentiles (violin plot), 1.5 × interquartile range (whiskers), and individual data are shown. Across the studies, a linear, time‐independent, PK profile was observed, and the PK of multiple doses of UCB0599 were predictable from single‐dose exposures. AUC, area under the plasma concentration–time curve; *C*_max_, maximum plasma concentration; PK, pharmacokinetics; MD, multiple dose; MD 180 mg at steady state (twice daily dosing 90 mg and includes data from UP0030 and UP0077); MD 360 mg at steady state (twice daily dosing 180 mg and includes data from UP0077 only); SD, single dose.

The shape of the plasma concentration–time profiles for the desmethyl and N‐oxide metabolites, including the elimination phases, were similar to those observed for the UCB0599 parent compound. However, compared with UCB0599, plasma exposures of the desmethyl metabolite were lower, and the apparent elimination half‐life was slightly longer (Table 3). The observed UCB0599 fraction unbound was not notably different between dose groups (Table 3). No chiral interconversion occurred (formation of the *S*‐enantiomer when the *R*‐enantiomer was dosed) as plasma levels of the *S*‐enantiomer were below the limit of quantification after the administration of steady‐state doses (180 or 360 mg/day) of the UCB0599 *R*‐enantiomer. PK results were consistent with those observed in HP studies with UCB0599 (Fig. 2).

## Discussion

This is the first report of clinical studies completed with an oral, brain‐penetrant, small‐molecule inhibitor of ASYN misfolding for the treatment of PD. UCB0599 is currently the only small molecule designed to target ASYN misfolding in phase 2 development.^10^, ^21^ In contrast with other ASYN‐focused agents in development, UCB0599 binds and stabilizes unfolded ASYN and therefore has a similar approach to that of tafamidis on transthyretin, shown to slow disease progression of hereditary transthyretin amyloidosis.^22^ In a preclinical model, chronic dosing of UCB0599 resulted in reduced aggregated ASYN levels (demonstrating target engagement), which has been shown to improve motor endpoints closely related to PD in transgenic mouse models.^16^ Antibody‐based ASYN‐related agents in development for PD typically bind extracellular aggregated ASYN to block the pathological spread from cell to cell, therefore acting later in the disease cascade.^23^

Across the doses assessed, UCB0599 demonstrated an acceptable safety/tolerability profile in HPs and participants with PD with age‐related comorbidities; the majority of AEs were mild‐to‐moderate in intensity, and no increase in the frequency/severity of TEAEs was observed with increasing doses of UCB0599. In the UP0030 study, HSRs were reported for 2 participants from the 180 mg/day group, both moderate in intensity. During UP0077, all participants with PD stayed in a research unit; this 4‐week observation period allowed closer monitoring of any changes in safety signals. Two HSRs were reported in UP0077; 1 additional rash event was reported in the 360 mg/day UCB0599 group. Therefore, HSR is an important identified risk that will continue to be monitored in subsequent studies with UCB0599.

Exposure data indicated that drug disposition was similar in HPs and participants with PD. No meaningful food effect on UCB0599 absorption was observed when PK properties of oral UCB0599 under fasted/fed conditions were compared. Food intake is expected to delay gastric emptying, thus impacting the time to maximum concentration of UCB0599, as demonstrated by the small increase in *t*_max_. Co‐administration of 200 mg of ITZ (a strong CYP3A4 inhibitor) had a significant effect on UCB0599 disposition, with an increase of more than twofold in UCB0599 plasma exposure, confirming that UCB0599 is a moderately sensitive CYP3A4 substrate. This interaction had no effect on the overall safety profile of UCB0599. Although long‐term safety effects cannot be extrapolated from these phase 1 data, phase 2 investigation, including dose‐exposure characteristics, is warranted.

Here, we showed that UCB0599 concentrations in the CSF increased linearly with increasing doses, building on a previous positron emission tomography study that demonstrated good brain penetration in the white and gray matter regions and an uptake rate consistent with rapid free distribution across the blood–brain barrier in HPs, after 360 mg UCB0599 administration.^11^ The apparent elimination half‐life of UCB0599 was consistent across all three studies (range: 9.3–13.1 hours). A linear, time‐independent, PK profile was observed, and the PK of multiple doses of UCB0599 were predictable from single‐dose exposures. *C*_max_ and AUC increased linearly across doses in HPs and participants with PD, and UCB0599 fraction unbound (~1.1%; measured ex‐vivo in plasma) was constant between dose groups.

In summary, 73 HPs and 21 participants with PD received single and/or multiple doses of UCB0599; the combination of an acceptable safety/tolerability profile and predictable PK supports the continued development of UCB0599 for slowing PD progression. A phase 2 study (ORCHESTRA; NCT04658186), evaluating the efficacy, safety, tolerability, and PK of oral UCB0599 in people with early‐stage PD, is underway.

## Author contributions

J.W.S: study conception (study design and protocol review) and organization (UP0030); statistical analysis: design, execution, and review; manuscript preparation: writing the first draft, review, and critique. P.B: study conception and execution (UP0077 and UP0078); manuscript preparation: writing the first draft, review, and critique. M.K.P: study conception (UP0077 and UP0078) and organization (UP0030); statistical analysis: design, execution, and review; manuscript preparation: writing the first draft, review, and critique. L.D: study conception, organization, and execution (UP0077 only); statistical analysis: review and critique; manuscript preparation: writing the first draft, review, and critique. F.‐X.M: study conception (study design and protocol review); manuscript preparation: writing the first draft, review, and critique. A.S: study conception, organization, and execution (except UP0030); manuscript preparation: writing the first draft, review, and critique. M.L: study conception (protocol amends UP0030), organization (UP0030), and execution (data collection, analysis, and interpretation—UP0030); manuscript preparation: review and critique. M.G: study conception (study design and protocol review), organization (UP0030), and execution (statistics/PK analysis and preparation of overview violin plots—UP0030); statistical analysis: design, execution, and review; manuscript preparation: writing the first draft, review, and critique. CD: study execution (UP0077 and UP0078); manuscript preparation: writing the first draft, review, and critique. A.‐L.B: study conception (UP0077 only); manuscript preparation: writing the first draft, review, and critique. M.B: study conception (study design and protocol review); manuscript preparation: review and critique. S.C: study conception, organization, and execution (except UP0030); statistical analysis: review and critique; manuscript preparation: writing the first draft, review, and critique. J.G: study conception (except UP0030), organization, and execution (UP0077 only); statistical analysis: review and critique; manuscript preparation: writing the first draft, review, and critique.

## Author Roles

1. Study:
  - Conception: A.S. (except UP0030); J.G. (except UP0030); A.‐L.B. and L.D. (UP0077 only); S.C. (except UP0030); M.K.P. and P.B. (UP0077 and UP0078); J.W.S., M.B., M.G., and F.‐X.M. (study design and protocol review); and M.L. (protocol amends UP0030)
  - Organization: A.S. (except UP0030); J.G. and L.D. (UP0077 only); S.C. (except UP0030); and M.K.P., J.W.S., M.G., and M.L. (UP0030)
  - Execution: A.S. (except UP0030), J.G. and L.D. (UP0077 only), S.C. (except UP0030), C.D. (UP0077 and UP0078), P.B. (UP0077 and UP0078), M.G. (statistics/PK analysis and preparation of overview violin plots—UP0030), M.L. (data collection, analysis, and interpretation—UP0030)
2. Statistical analysis
  - Design: M.K.P., J.W.S., and M.G.
  - Execution: M.K.P., J.W.S., and M.G.
  - Review and critique: J.G., L.D., S.C., M.K.P., J.W.S., and M.G.
3. Manuscript preparation
  - Writing first draft: all authors except M.B. and M.L.
  - Review and critique: all authors

## Funding agency

These studies were funded by UCB Pharma.

## Full financial disclosures of all authors for the preceding 12 months

All authors were employees of UCB Pharma during the course of these studies and may hold/have access to stock options.

## Supporting information


**APPENDIX S1**. Supplementary Information

## Data Availability

Due to the small sample sizes in these trials, individual participant‐level data cannot be adequately anonymized as there is a reasonable likelihood that individual participants could be re‐identified. For this reason, data from this trial cannot be shared.
