# Supplementary material for: Phase 1/1b Studies of UCB0599, an Oral Inhibitor of α‐Synuclein Misfolding, Including a Randomized Study in Parkinson's Disease
Source: Mov Disord. 2022 Aug 12;37(10):2045–56. doi: 10.1002/mds.29170 (PMC9804489; doi:10.1002/mds.29170)
Supplement: Supplementary file 1 — APPENDIX S1. Supplementary Information [file MDS-37-2045-s001.pdf]

Supplementary Information

Phase 1/1b studies of UCB0599, an oral inhibitor of α-synuclein misfolding, including a randomized study in Parkinson's Disease

UCB0599 Phase 1/1b results

Johan Willem Smit, PhD[1], Peter Basile, MBBS FFPM[2], Maria Key Prato, MSc[1], Laurent Detalle, PhD[1], François-Xavier Mathy, Pharm PhD[1], Astrid Schmidt, Dr. rer. nat[3], Marianna Lalla, MD, PhD, MPH[2], Massimiliano Germani[1], Coralie Domange, MSc[4], Anja-Leona Biere, PhD[4], Massimo Bani, PhD[1*], Stan Carson, PharmD[6], Just Genius, MD[1†]

[1]UCB Pharma, Braine-l'Alleud, Belgium; [2]UCB Pharma, Slough, UK; [3]UCB Pharma, Monheim am Rhein, Germany; [4]UCB Pharma, Brussels, Belgium; [5]Bergmapharm Consulting, Verona, Italy; [6]UCB Pharma, North Carolina, USA

*Current affiliation: Bergmapharm Consulting, Verona, Italy

†Current affiliation: Genius Biotech Solutions, Ltd, Victoria, Gozo Island, Republic of Malta

Corresponding author:

Stan Carson

Address: UCB Pharma, 4000 Paramount Parkway, Suite 200, Morrisville, NC 27560, USA

Telephone: +1-919-698-4990

Email: Stan.Carson@ucb.com

Supplementary Methods:

Ethical conduct

These studies were conducted in accordance with the current version of the applicable regulatory and International Council for Harmonization-Good Clinical Practice requirements, the ethical principles with origins in the Declaration of Helsinki, and the local laws of the countries involved. Study information was provided in text and verbally by the investigator; informed consent forms were signed and dated by the participants.

UP0030: ***two-part Phase 1 study evaluating safety/tolerability, PK and the effect of food and itraconazole (ITZ) on UCB0599***

Sample size and randomization

A sample size of 58 participants was considered sufficient to meet the primary objectives, and 14 participants were considered sufficient to determine bioequivalence with food at a power of 80%, and a true mean ratio around 1.

The study investigator determined subject eligibility for the study and randomization numbers were manually allocated to eligible participants, and treatment was dispensed according to the randomization schedule by a blinded pharmacist. Sponsor and investigator-site personnel involved in the study were blinded to treatment assignment, and placebo capsules generated to match UCB0599 to maintain blinding.

UP0078*: **Phase 1 study evaluating the effect of food and ITZ on UCB0599***

Sample size and randomization

Required sample size was estimated using nQuery Version 7 software (Statistical Solutions Ltd., Cork Ireland), and confidence intervals (CI) were estimated using R Version 3.3.2. A sample size of 20 was required for the bioequivalence food effect study, to conclude bioequivalence with a power of 90%,

and a true mean fed/fasted AUC from Time 0 to infinity ($AUC_{0\text{-inf}}$) ratio of 1.1. The mean $AUC_{0\text{-inf}}$ ratio with/without ITZ was expected to be 1.25, with an expected 90% CI of 1.17–1.34 at this sample size.

Randomization numbers were computer generated and the randomization list was programed by the contract research organization. The study investigator was responsible for the enrolment and randomization of eligible participants; randomization was assigned at the site in an unblinded fashion and entered into the electronic case report form.

UP0077: Phase 1b study evaluating safety/tolerability and multi-dose PK of UCB0599

Sample size and randomization

It was expected that ~39 participants would be screened for study entry, and 27 would complete the study, to provide adequate exposure information.

To enroll a participant, the investigator contacted the interactive response technology (IRT) provider (Parexel®, Massachusetts, USA) who allocated a participant identification number. To randomize participants, the investigator contacted the IRT provider and participants were assigned to receive UCB0599 or placebo, according to a predetermined randomization schedule; randomization numbers were tracked via IRT. Participants, investigators, sponsor and study-site staff were blinded to treatment allocation. Placebo capsules were generated to resemble UCB0599 to maintain blinding.

Adverse events of special interest

An adverse event of special interest (AESI) was defined as any adverse event that the regulatory authority mandated be reported on an expediated basis regardless of seriousness, expectedness or relatedness to UCB0599. For UP0030 the following events were considered AESI: significant change(s) in laboratory findings that were suggestive of renal toxicity (e.g. significant elevation of serum creatinine or urea, signs of significant albuminuria, or renal injury biomarkers results adjudicated to be clinically relevant); and potential Hy's Law, defined as ≥ 3x the upper limit of

normal (ULN) alanine aminotransferase or aspartate aminotransferase with coexisting ≥ 2x ULN total bilirubin in the absence of ≥ 2x ULN alkaline phosphatase, with no alternative explanation for the biochemical abnormality (always to be reported as an AESI without waiting for any additional etiologic investigations to have been concluded). HSR was included as an AESI, instead of an adverse event of special monitoring, in a protocol amendment on the 25 June 2018.

For UP0078 and UP0077 the following events were considered AESIs: potential Hy's Law (as defined above) and hypersensitivity reactions (HSR), including symptoms commonly associated with HSR, such as anaphylaxis and angioedema.

TABLE S1. UP0077 (Phase 1b study in participants with PD) previous and ongoing medical conditions (MedDRA preferred term) and concomitant medications (reported by ≥2 participants)

	UCB0599	UCB0599	All UCB0599	Placebo
	180 mg/day	360 mg/day		
n (%)	n=7	n=14	n=21	n=10
**Previous and ongoing medical conditions**	**7 (100)**	**14 (100)**	**21 (100)**	**10 (100)**
Presbyopia	1 (14.3)	2 (14.3)	3 (14.3)	4 (40.0)
Cataract	0	2 (14.3)	2 (9.5)	0
Myopia	0	1 (7.1)	1 (4.8)	2 (20.0)
Constipation	1 (14.3)	4 (28.6)	5 (23.8)	2 (20.0)
Gastroesophageal reflux disease	1 (14.3)	4 (28.6)	5 (23.8)	2 (20.0)
Seasonal allergy	1 (14.3)	3 (21.4)	4 (19.0)	1 (10.0)
Drug hypersensitivity	2 (28.6)	0	2 (9.5)	1 (10.0)
Appendicitis	0	1 (7.1)	1 (4.8)	2 (20.0)
Tonsillitis	1 (14.3)	0	1 (4.8)	3 (30.0)
Hypercholesterolemia	1 (14.3)	3 (21.4)	4 (19.0)	1 (10.0)
Vitamin D deficiency	0	1 (7.1)	1 (4.8)	2(20.0)
Arthralgia	0	3 (21.4)	3 (14.3)	0
Muscle Spasms	0	3 (21.4)	3 (14.3)	0
Myalgia	1 (14.3)	2 (14.3)	3 (14.3)	0
Rotator cuff syndrome	0	2 (14.3)	2 (9.5)	0
Headache	1 (14.3)	3 (21.4)	4 (19.0)	0
Restless legs syndrome	0	2 (14.3)	2 (9.5)	0
Transient ischemic attack	0	0	0	2 (20.0)
Depression	1 (14.3)	5 (35.7)	6 (28.6)	2 (20.0)
Anxiety	1 (14.3)	2 (14.3)	3 (14.3)	2 (20.0)

Insomnia	1 (14.3)	2 (14.3)	3 (14.3)	2 (20.0)
Nephrolithiasis	0	2 (14.3)	2 (9.5)	0
Benign prostatic hyperplasia	1 (14.3)	2 (14.3)	3 (14.3)	2 (20.0)
Erectile dysfunction	0	2 (14.3)	2 (9.5)	2 (20.0)
Asthma	1 (14.3)	4 (28.6)	5 (23.8)	0
Hypertension	3 (42.9)	2 (14.3)	5 (23.8)	2 (20.0)
**Prior medications**	**5 (71.4)**	**14 (100)**	**19 (90.5)**	**10 (100)**
**Concomitant medications**	**7 (100)**	**14 (100)**	**21 (100)**	**10 (100)**
Docusate sodium	0	3 (21.4)	3 (14.3)	1 (10.0)
Polycarbophil calcium	0	2 (14.3)	2 (9.5)	0
Pantoprazole	0	2 (14.3)	2 (9.5)	0
Magnesium	0	2 (14.3)	2 (9.5)	1 (10.0)
Cholecalciferol	0	3 (21.4)	3 (14.3)	1 (10.0)
Vitamin B complex	0	2 (14.3)	2 (9.5)	1 (10.0)
Atorvastatin	1 (14.3)	1 (7.1)	2 (9.5)	2 (20.0)
Tamsulosin Hydrochloride	0	2 (14.3)	2 (9.5)	0
Ibuprofen	3 (42.9)	5 (35.7)	8 (38.1)	1 (10.0)
Clonazepam	0	3 (21.4)	3 (14.3)	1 (10.0)
Sinemet	4 (57.1)	14 (100)	18 (85.7)	8 (80.0)
Amantadine	2 (28.6)	3 (21.4)	5 (23.8)	2 (20.0)
Entacapone	0	2 (14.3)	2 (9.5)	0
Pramipexole dihydrochloride	0	2 (14.3)	2 (9.5)	0
Rasagiline mesylate	0	2 (14.3)	2 (9.5)	0
Ropinirole	0	2 (14.3)	2 (9.5)	1 (10.0)
Pramipexole	1 (14.3)	0	1 (4.8)	2 (20.0)
Rasagiline	0	0	0	3 (30.0)
Selegiline	0	0	0	2 (20.0)

Paracetamol	5 (71.4)	3 (21.4)	8 (38.1)	0
Cannabidiol	0	2 (14.3)	2 (9.5)	0
Fexofenadine hydrochloride	0	2 (14.3)	2 (9.5)	0

n, number of participants; MedDRA, Medical Dictionary for Regulatory Activities; PD, Parkinson's disease

TABLE S2. UP0030 (Phase 1 study in HP) incidence of TEAEs by MedDRA preferred term (reported by ≥2 participants)

n (%) [#]	UCB0599 90 mg n=9	UCB0599 180 mg n=17	UCB0599 360 mg n=18	UCB0599 450 mg n=9	UCB0599 180 mg/day n=17	Total UCB0599 N=45	Placebo n=14
Any TEAE	8 (89) [17]	10 (59) [15]	13 (72) [25]	4 (44) [10]	7 (41) [22]	30 (67) [89]	9 (64) [27]
Diarrhea	0	0	2 (11) [2]	1 (11) [1]	0	3 (7) [3]	0
Puncture site pain	2 (22) [2]	2 (12) [2]	2 (11) [2]	0	2 (12) [2]	6 (13) [8]	2 (14) [3]
Fatigue	0	0	2 (11) [3]	0	0	2 (4) [3]	0
Drug hypersensitivity	0	0	0	0	2 (12) [3]	2 (4) [3]	0
Nasopharyngitis	0	0	0	1 (11) [1]	1 (6) [1]	2 (4) [2]	2 (14) [2]
Postlumbar puncture syndrome	2 (22) [2]	0	3 (17) [4]	1 (11) [1]	0	6 (13) [7]	2 (14) [3]
Myalgia	2 (22) [2]	0	0	0	1 (6) [1]	3 (7) [3]	1 (7) [1]
Headache	1 (11) [1]	0	1 (6) [1]	1 (11) [1]	2 (12) [2]	5 (11) [5]	1 (7) [1]
Presyncope	1 (11) [1]	1 (6) [1]	0	0	0	1 (2) [2]	2 (14) [2]

Across the doses assessed, UCB0599 demonstrated an acceptable safety/tolerability profile in HP; the majority of AEs were mild–moderate in intensity, and no increase in the frequency/severity of TEAEs was observed with increasing doses of UCB0599.

HP, healthy participants; n, number of patients reporting a TEAE in that category; [#], number of TEAEs; MedDRA, Medical Dictionary for Regulatory Activities; TEAE, treatment-emergent adverse event

TABLE S3. UP0077 (Phase 1b study in participants with PD) incidence of TEAEs by MedDRA preferred term (reported by ≥2 participants)

n (%) [#]	UCB0599 180 mg/day n=7	UCB0599 360 mg/day n=14	Total UCB0599 N=21	Placebo n=10
Any TEAE	6 (86) [19]	11 (79) [36]	17 (81) [55]	7 (70) [19]
Post-lumbar puncture syndrome	0	2 (14) [2]	2 (10) [2]	0
Glomerular filtration rate decreased	0	2 (14) [3]	2 (10) [3]	2 (20) [2]
Syncope	0	0	0	2 (20) [2]
Headache	5 (71) [8]	2 (14) [2]	7 (33) [10]	2 (20) [2]
Hypotension	0	2 (14) [5]	2 (10) [5]	0

Across the doses assessed, UCB0599 demonstrated an acceptable safety/tolerability profile, in participants with PD with age-related comorbidities; the majority of AEs were mild–moderate in intensity, and no increase in the frequency/severity of TEAEs was observed with increasing doses of UCB0599.

n, number of participants reporting a TEAE in that category; [#], number of TEAE; MedDRA, Medical Dictionary for Regulatory Activities; PD, Parkinson's disease; TEAE, treatment-emergent adverse event

TABLE S4. UP0077 (Phase 1b study in participants with PD) TEAEs considered related to study medication by MedDRA preferred term (reported by ≥2 participants)

n (%)	UCB0599 180 mg/day n=7	UCB0599 360 mg/day n=14	Total UCB0599 N=21	Placebo n=10
Any related TEAE	4 (57)	5 (36)	9 (43)	3 (30)
Glomerular filtration rate decreased	0	2 (14)	2 (10)	2 (20)
Headache	3 (43)	2 (14)	5 (24)	0
Hypotension	0	2 (14)	2 (10)	0

Across the doses assessed, UCB0599 demonstrated an acceptable safety/tolerability profile, in participants with PD with age-related comorbidities; the majority of AEs were mild–moderate in intensity, and no increase in the frequency/severity of TEAEs was observed with increasing doses of UCB0599.

n, number of participants reporting a TEAE in that category; MedDRA, Medical Dictionary for Regulatory Activities; PD, Parkinson's disease; TEAE, treatment-emergent adverse event

TABLE S5. UP0078 (Phase 1 study in HP) pharmacokinetic parameters of UCB0599 and its metabolites

	Statistics	Treatment group	n	UCB0599	Desmethyl metabolite	N-oxide metabolite
$AUC_{(0–t)}$, h*ng/mL	Geometric mean	Fasted	22	5980 (33.4)	262.6 (43.2)	5348 (41.0)
	(CV%)	Fed	22	6515 (37.0)	289.0 (50.3)	5757 (46.3)
		ITZ	21	16,520 (25.2)	1447 (35.5)	18,800 (33.5)
C_{max}, ng/mL	Geometric mean	Fasted	22	737.8 (42.8)	16.24 (35.0)	361.8 (33.6)
	(CV%)	Fed	22	770.8 (32.8)	17.50 (43.4)	416.6 (37.0)
		ITZ	21	964.4 (55.9)	24.59 (40.8)	692.3 (45.7)
t_{max}, h	Geometric mean	Fasted	22	1.5 (1.0–3.0)	1.5 (1.0–6.0)	3.0 (2.0–8.0)
	(CV%)	Fed	22	3.0 (0.5–5.0)	3.5 (1.0–10.0)	4.0 (1.5–8.0)
		ITZ	21	2.0 (1.0–24.1)	10.0 (2.0–48.1)	5.0 (3.0–24.0)
Half-life, h	Geometric mean	Fasted	22	9.3 (18.4)	13.6 (13.1)	9.2 (18.4)
	(CV%)	Fed	22	9.6 (25.4)	13.2 (18.9)	9.7 (25.4)
		ITZ	21	15.9 (14.8)	31.6 (14.7)	16.6 (14.9)

The Cmax and AUC GeoMean for UCB0599 were comparable under fed and fasted conditions.

$AUC_{(0–t)}$, area under the plasma concentration-time curve from 0 to last quantifiable concentration; C_{max}, maximum plasma concentration; CV, coefficient of variation; HP, healthy participants; h, hours; t_{max}, time of maximum concentration

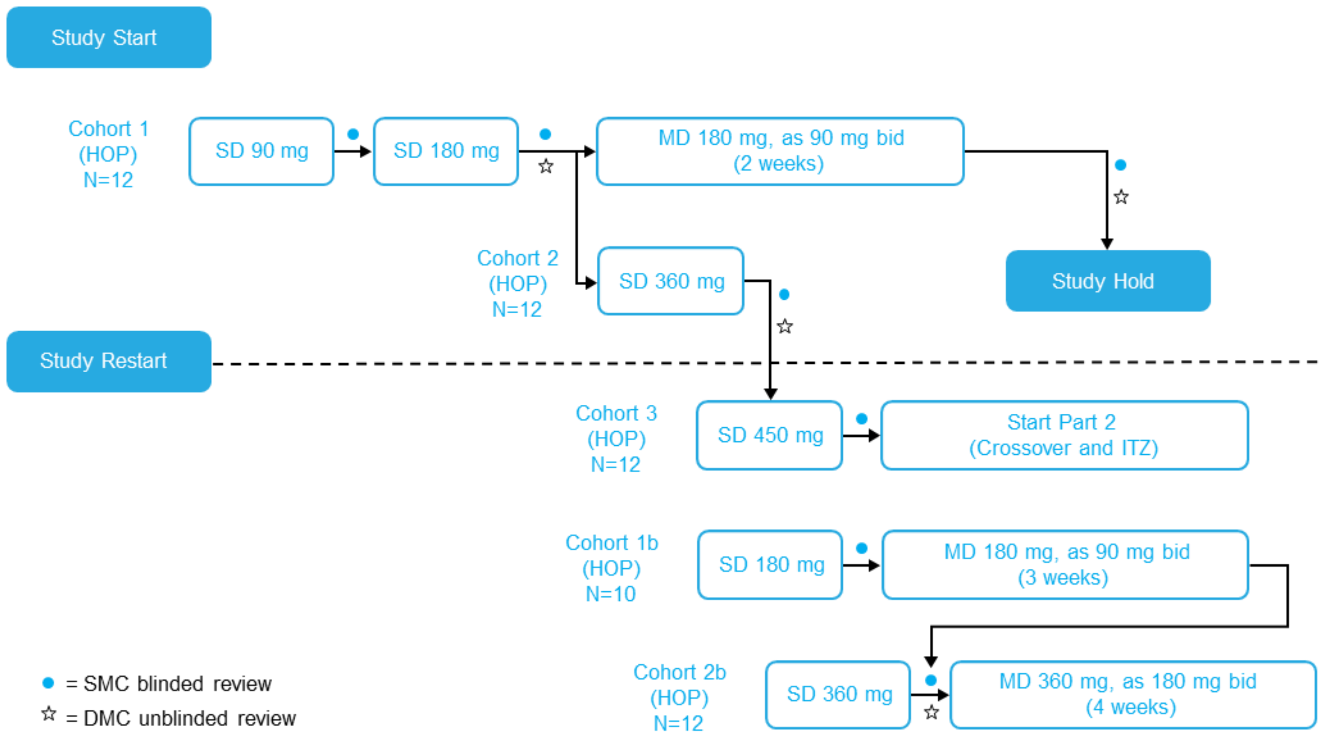

FIG S1A. UP0030, Phase 1, Part 1 single- and multiple-ascending-dose study evaluating safety tolerability and PK of UCB0599 in HOP.

bid, twice daily; DMC, Data Monitoring Committee; HOP, healthy older participants; ITZ, itraconazole; MD, multiple dose; SD, single dose; SMC, Safety Monitoring Committee

Note: Does not reflect sentinel pair and staggered approach to dosing subjects in Cohorts 1, 2, 2b, and 3.

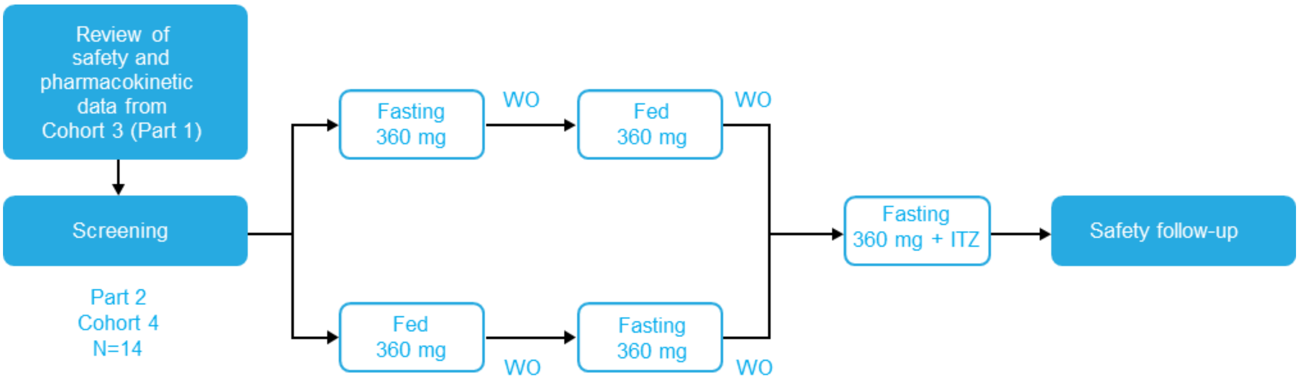

FIG S1B. UP0030, Phase 1, Part 2, 2-way crossover study with a third treatment period, evaluating food and drug–drug interactions in HP.

HP, healthy participants; WO, washout period of 7–14 days

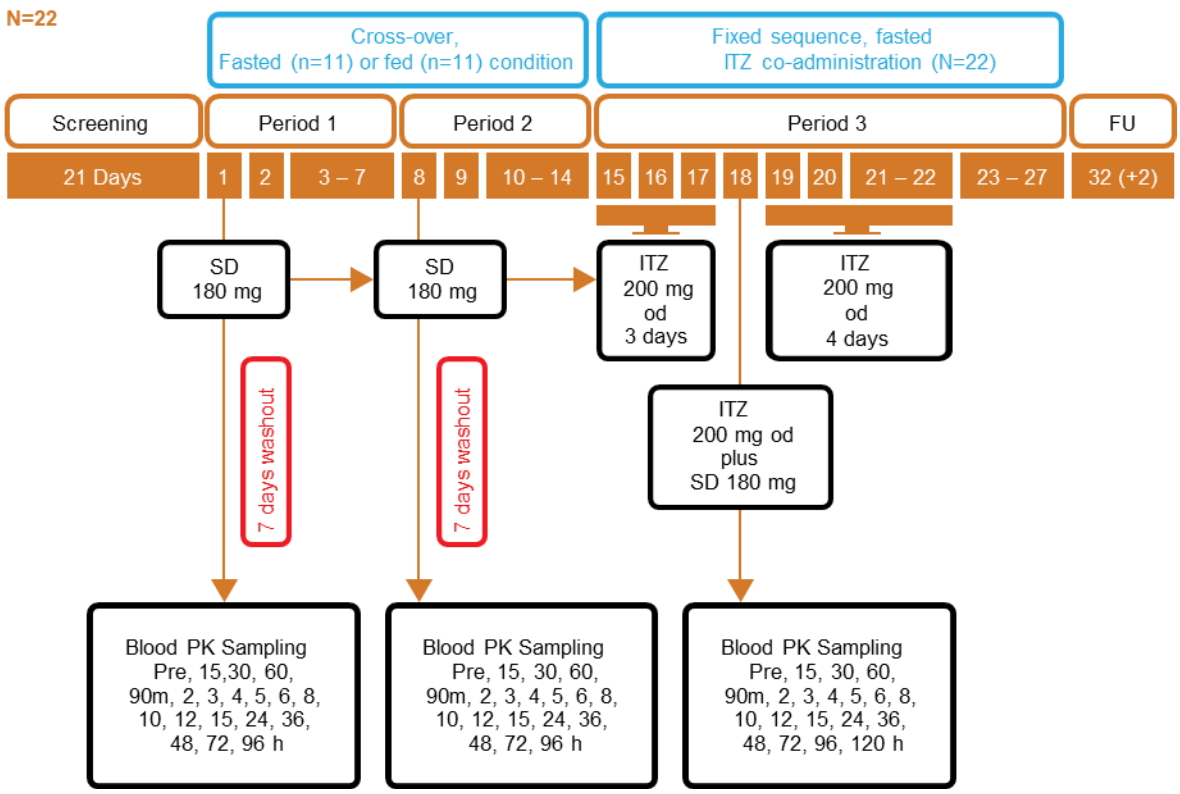

FIG S2. UP0078, Phase 1 study in HP evaluating the effect of food and ITZ on the bioavailability and disposition of UCB0599, respectively.

In this study, a randomized, two-way crossover design with a third fixed-treatment period was used.

FU, follow up; h, hours; HP, healthy participants; ITZ, itraconazole; m, minutes; od, once per day; PK, pharmacokinetics; pre, predose; SD, single dose

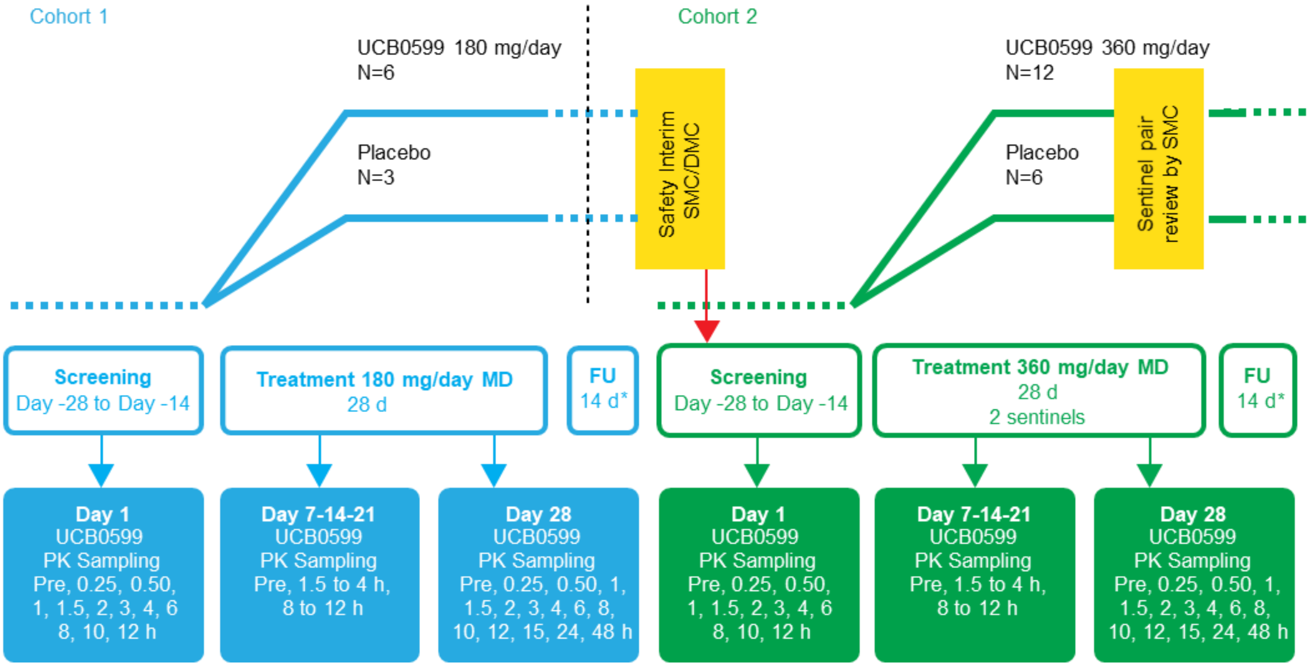

FIG S3. UP0077, Phase 1b study evaluating safety, tolerability and multi-dose PK of UCB0599 in participants with PD.

*Participants stayed in clinical research units/specialized medical centers for the entire treatment period and 48 hours after the last dose (which is included in the FU period)

d, days; DMC, Data Monitoring Committee; FU, follow up; MD, multiple doses; PD, Parkinson's disease; PK, pharmacokinetic; SMC, Safety Monitoring Committee

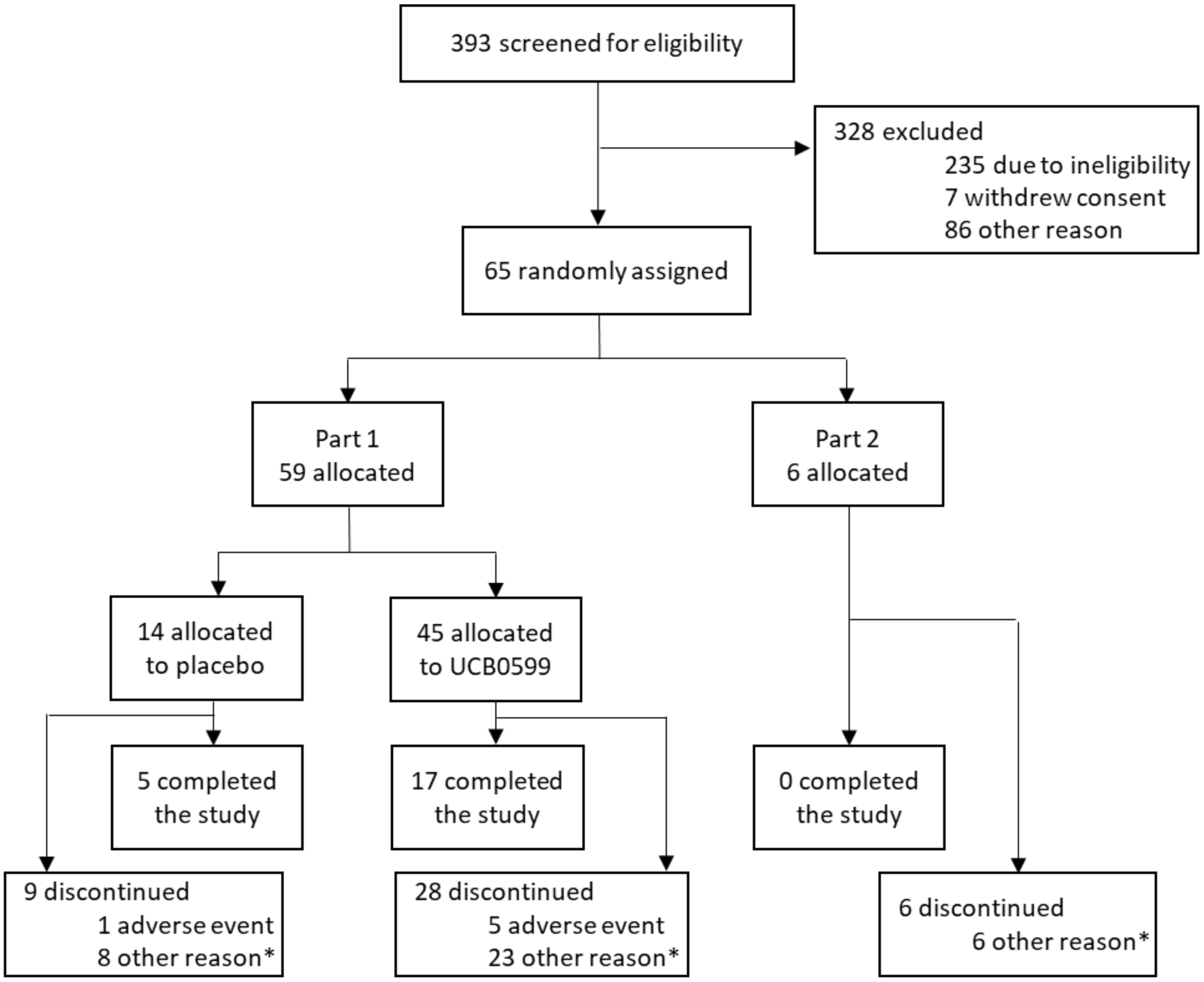

*Due to study termination

Fig S4: UP0030 (Phase 1 study in HPs): CONSORT diagram.

Between March 08, 2017 and May 18, 2017, 65 HPs were randomized to receive UCB0599.

HP, healthy participant

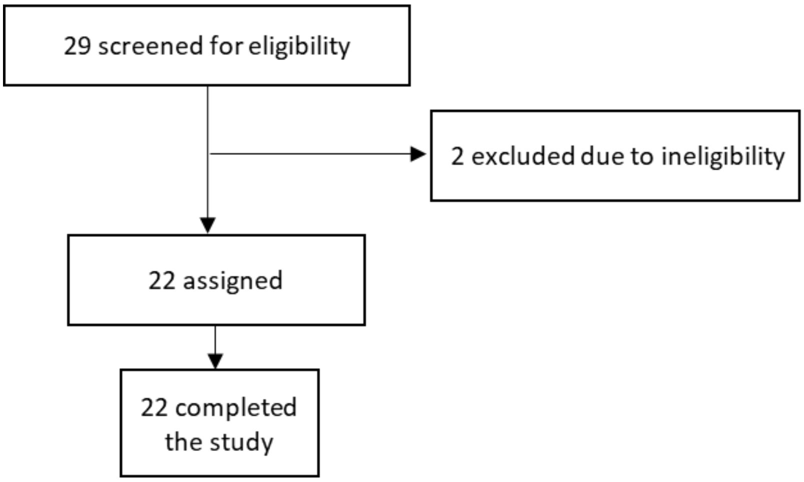

Fig S5: UP0078 (Phase 1 study in HPs): CONSORT diagram.

Between October 29, 2019 and January 09, 2020, 22 HPs were randomized to receive UCB0599, and all participants completed the study.

HP, healthy participant

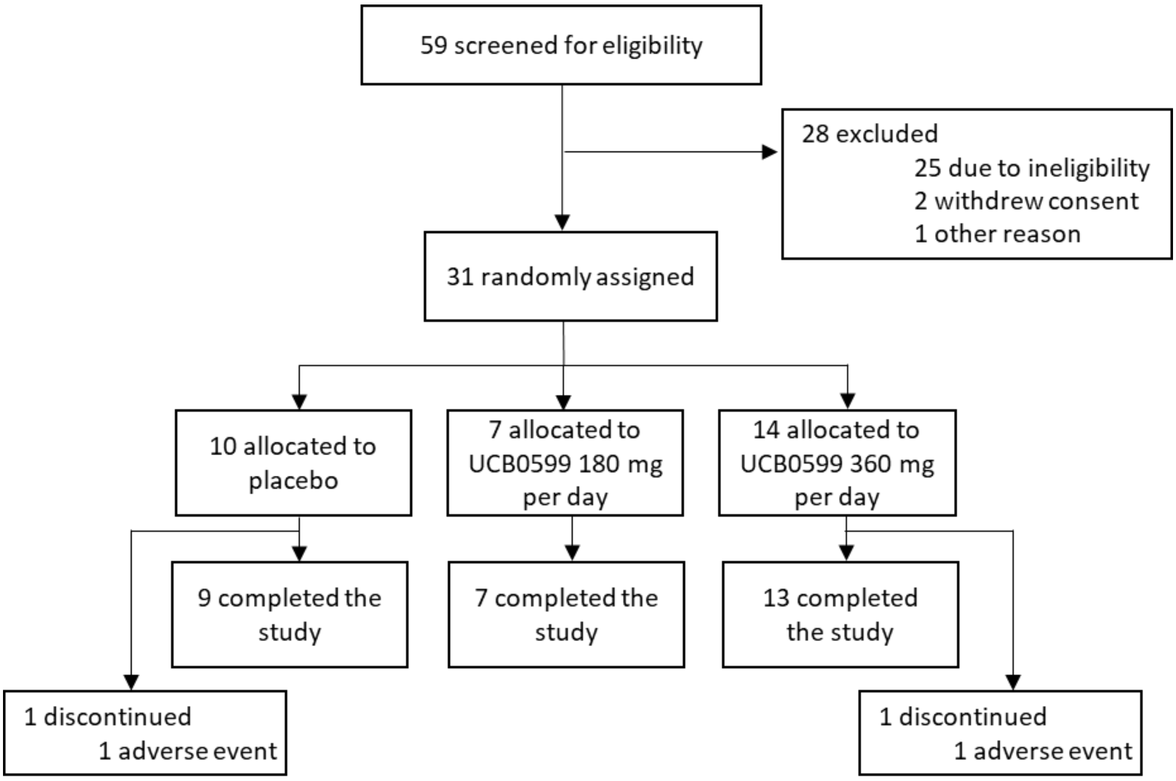

Fig S6: UP0077 (Phase 1b study in participants with PD): CONSORT diagram.

Between May 06, 2019, and February 19, 2020, 31 participants with PD were randomized to receive UCB0599 or placebo.

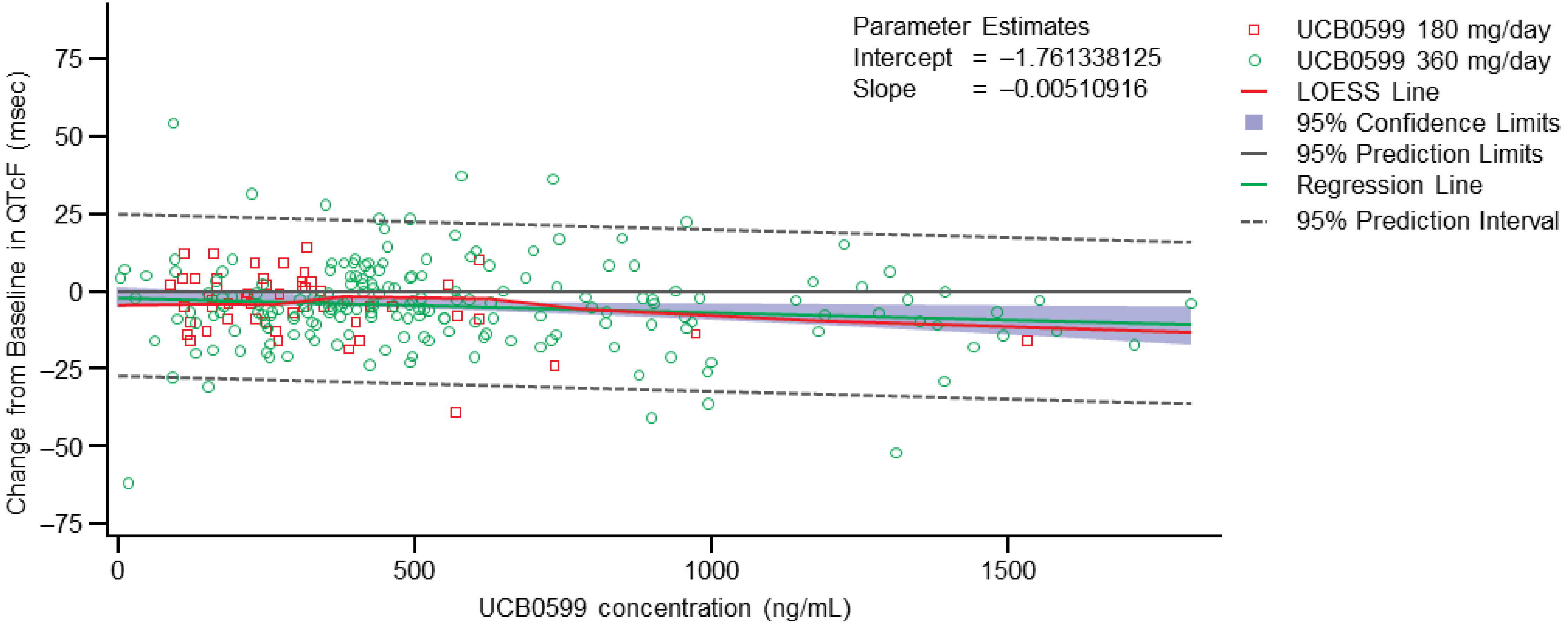

Fig S7: Regression analysis of delta corrected QT interval by Fredericia versus UCB0599 plasma concentration in UP0077 (Phase 1b study in participants with PD).

No clinically relevant treatment-related pattern was observed for QTcF intervals.

LOESS, locally estimated scatterplot smoothing; QTcF, QT interval corrected using Fridericia's correction

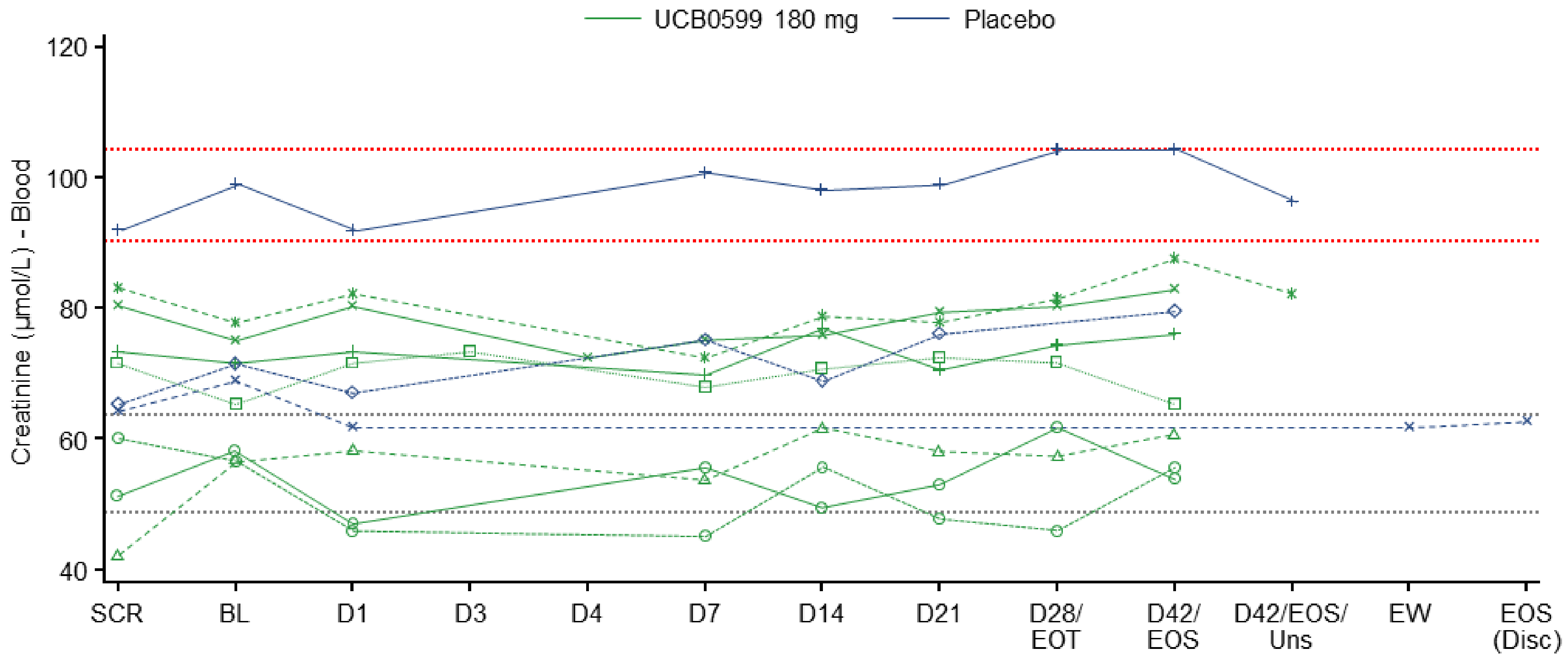

Fig S8a: Serum creatine (µmol/L) over time for Cohort 1 participants receiving UCB0599 180 mg/day or placebo in UP0077 (Phase 1b study in participants with PD).

Serum creatinine reference ranges are depicted by the dotted gray (lower) and red (upper) lines; the ranges are 48.62– 90.17 µmol/L for females and 63.65–104.31 µmol/L for males. No clinically relevant treatment-related pattern was observed for plasma creatinine trajectories.

BL, baseline; D, Day; Disc, discontinuation; EOS, end of study; EOT, end of treatment; EW, early withdrawal; SCR, screening; Uns, unscheduled

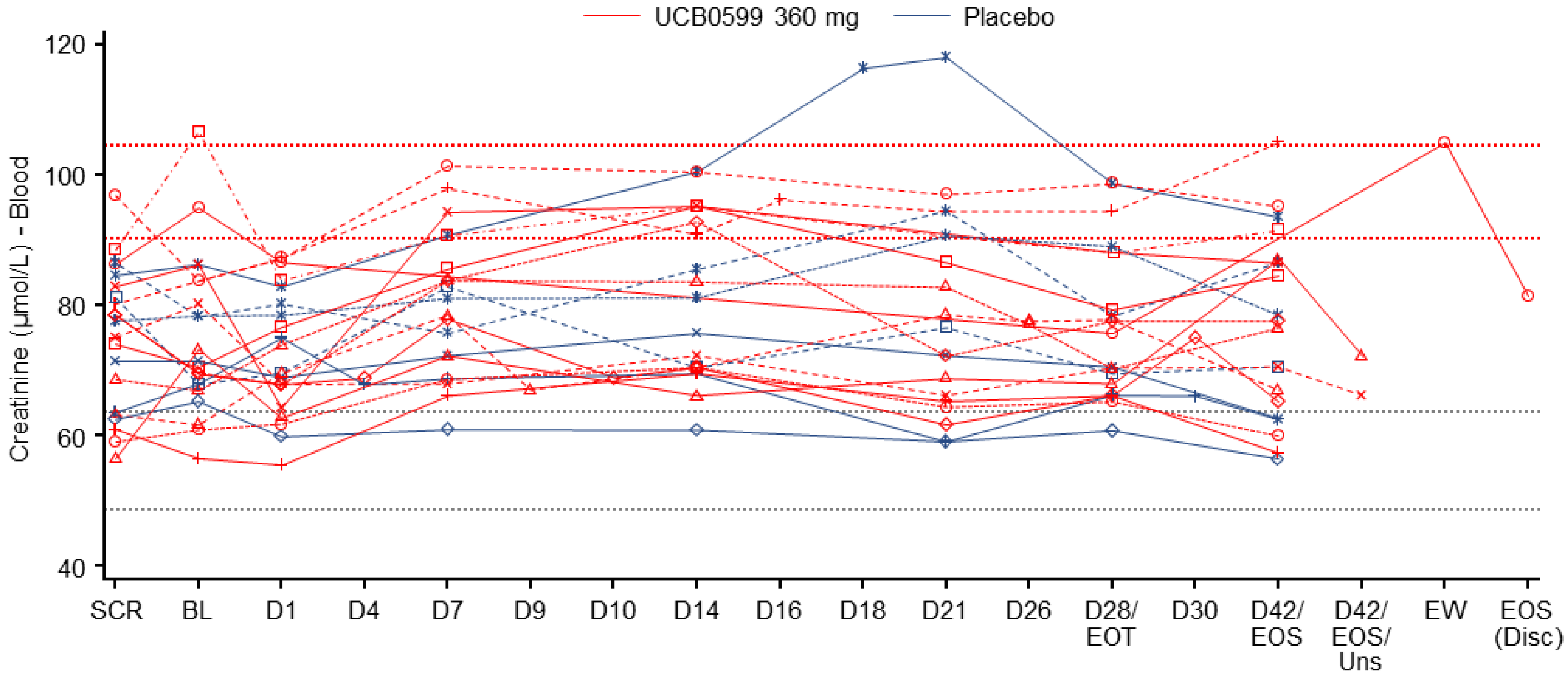

Fig S8b: Serum creatinine (µmol/L) over time for participants in Cohort 2 receiving UCB0599 360 mg/day or placebo in UP0077 (Phase 1b study in participants with PD).

Serum creatinine reference ranges are depicted by the dotted gray (lower) and red (upper) lines; the ranges are 48.62– 90.17 µmol/L for females and 63.65–104.31 µmol/L for males. No clinically relevant treatment-related pattern was observed for plasma creatinine trajectories.

BL, baseline; D, Day; Disc, discontinuation; EOS, end of study; EOT, end of treatment; EW, early withdrawal; SCR, screening; Uns, unscheduled

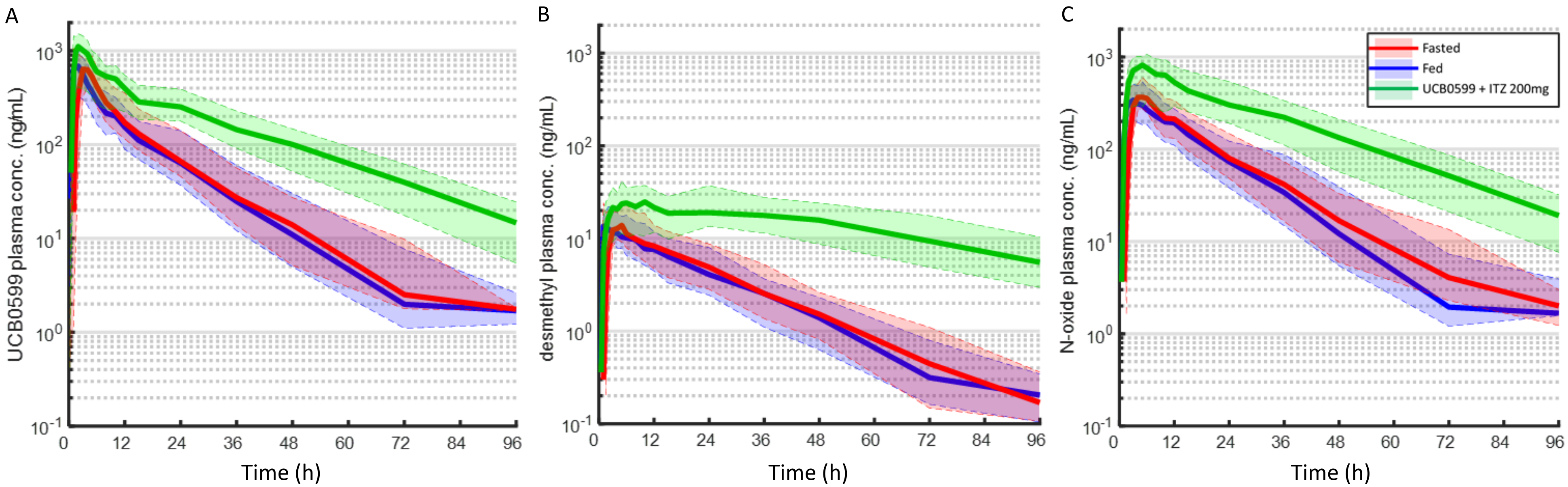

Fig S9: Observed UCB0599 (A), desmethyl (B) and N-oxide metabolite (C) plasma profiles in fasted and fed conditions, and in combination with 200 mg ITZ, following single oral administration of UCB0599 180 mg, in UP0078 (Phase 1 study in HP).

Data shown are median profiles (solid lines) and participant variability (shaded areas delimited by 10th and 90th percentiles). Co-administration of UCB0599 and ITZ resulted in increased (peak) plasma concentrations for UCB0599; effects observed on metabolite concentration-time profiles were consistent with observations for the UCB0599 parent molecule.

Conc., concentration; h, hours; HP, healthy participants; ITZ, itraconazole
